# MicroRNA (miRNA)-to-miRNA Regulation of Programmed Cell Death 4 (PDCD4)

**DOI:** 10.1128/MCB.00086-19

**Published:** 2019-08-27

**Authors:** Pamela Ajuyah, Meredith Hill, Alireza Ahadi, Jing Lu, Gyorgy Hutvagner, Nham Tran

**Affiliations:** a School of Life Sciences, Faculty of Science, University of Technology Sydney, Sydney, NSW, Australia; b School of Biomedical Engineering, Faculty of Engineering and Information Technology, University of Technology Sydney, Sydney, NSW, Australia; c The Sydney Head and Neck Cancer Institute, Sydney Cancer Centre, Royal Prince Alfred Hospital, Sydney, NSW, Australia

**Keywords:** cancer biology, cooperative regulation, gene silencing, head and neck squamous cell carcinoma, HNSCC, microRNA, miRNA

## Abstract

The regulation of tumor suppressor genes by microRNAs (miRNAs) is often demonstrated as a one-miRNA-to-one-target relationship. However, given the large number of miRNA sites within a 3′ untranslated region (UTR), most targets likely undergo miRNA cooperation or combinatorial action. Programmed cell death 4 (PDCD4), an important tumor suppressor, prevents neoplastic events and is commonly downregulated in cancer. This study investigates the relationship between miRNA 21 (miR-21) and miR-499 in regulating PDCD4. This was explored using miRNA overexpression, mutational analysis of the PDCD4 3′ UTR to assess regulation at each miRNA site, and 50% inhibitory concentration (IC_50_) calculations for combinatorial behavior. We demonstrate that the first miR-499 binding site within PDCD4 is inactive, but the two remaining sites are both required for PDCD4 suppression. Additionally, the binding of miR-21 to PDCD4 influenced miR-499 activity through an increase in its silencing potency and stabilization of its mature form. Furthermore, adjoining miRNA sites more than 35 nucleotides (nt) apart could potentially regulate thousands of 3′ UTRs, similar to that observed between miR-21 and miR-499. The regulation of PDCD4 serves as a unique example of regulatory action by multiple miRNAs. This relationship was predicted to occur on thousands of targets and may represent a wider mode of miRNA regulation.

## Abstract (precis)

The regulation of tumor suppressor genes by microRNAs (miRNAs) is often demonstrated as a one-miRNA-to-one-target relationship. However, given the large number of miRNA sites within a 3′ untranslated region (UTR), most targets likely undergo miRNA cooperation or combinatorial action. Programmed cell death 4 (PDCD4), an important tumor suppressor, prevents neoplastic events and is commonly downregulated in cancer.

## INTRODUCTION

Most human cancers represent an uncoupling of the gene expression pathways that oversee cellular homeostasis. Mammalian cells express tumor suppressor genes and provide a protective network to guard against transformation events. One such tumor suppressor is programmed cell death 4 (PDCD4), which protects the cell from early neoplastic changes (1) and is frequently downregulated in many cancers (2–6). There exists a complicated network of direct and indirect mechanisms controlling the expression of tumor suppressors such as PDCD4 (7). In recent years, microRNAs (miRNAs), members of the noncoding RNA (ncRNA) family, have been shown to be potent regulators of gene expression (8, 9). Small ncRNAs, including miRNAs, are processed through a series of enzymatic steps to produce a 22-nucleotide (nt) miRNA duplex (10). One strand of this duplex is loaded onto a silencing complex which directly associates with target mRNAs to abrogate translation.

Typically, mammalian miRNAs bind to the 3′ untranslated region (UTR) of a target mRNA, with most studies describing a one-miRNA-to-one-target model. These results have been largely driven by the prudent use of cell models to deliver one miRNA mimic and measure the regulation of a single target gene. However, the 3′ UTR usually contains several hundred miRNA binding sites. Therefore, it stands to reason that different miRNAs must partake in some manner of combinatorial regulation to perform gene regulation. However, the mechanism behind the coregulation of tumor suppressor genes by multiple miRNAs is not fully understood.

In most situations, miRNA coregulation of a target can occur independently or in a cooperative manner. Independent binding takes place when several different miRNAs regulate the same target at different locations to confer a modest additive effect. In contrast, cooperativity is driven by the binding of miRNAs at adjoining sites and results in the amplification of regulatory potency. The difference between independent and cooperative regulation largely relies upon the distance between miRNA binding sites. Several biochemical studies have now determined that adjoining miRNA sites separated by 13 to 40 nt display functional cooperativity (11–14). Therefore, any deviation from this distance would likely shift the miRNA to an independent regulatory role.

This notion of miRNA cooperativity has been computationally explored on a genome-wide scale. There is a statistical enrichment for adjoining miRNA sites (15 to 26 nt apart) in the human transcriptome (15). It has been further suggested that 17,259 human genes could be regulated in a cooperative fashion by 29,000 distinct miRNA pairs (16). Importantly, an enrichment of adjoining binding sites is observed for those miRNAs involved in regulating disease processes. The scope of this coregulation underscores a widespread fundamental mechanism for miRNA-mediated silencing. Despite the overwhelming predictive proof of the cooperative action of miRNAs, most miRNA target evaluation studies focus only on the role of a single miRNA in the regulation of a targeted mRNA.

There are approximately 100 miRNA binding sites on the 3′ UTR of PDCD4, but only a select few have been experimentally validated. The main contributor, miRNA 21 (miR-21), can act independently to regulate the expression of PDCD4 (17, 18). When tested individually, it was also demonstrated that miR-182 (19), miR-183 (20), miR-320a (21), miR-141 (22), and miR-499 (23, 24) regulate PDCD4, but it is not known if these miRNAs display cooperativity. Since both miR-21 and miR-499 have been shown to regulate the expression of PDCD4 in a diverse range of cancers, we asked whether these miRNAs interact in their regulation of PDCD4. The binding sites for these two miRNAs are highly conserved, with three miR-499 sites, at nt 17 to 23, nt 467 to 473, and nt 533 to 539, and one miR-21 site, at nt 242 to 249, within the PDCD4 3′ UTR.

Our study attempts to investigate the relationship between miR-21, miR-499, and the regulation of PDCD4. We aim to determine whether these sites are dependent on each other and how this affects the level of target regulation. Currently, there are limited studies describing the action of multiple miRNAs on PDCD4. These findings contribute to a further understanding of PDCD4 regulation but also expand our understanding of the possibility of miRNAs working together to elicit target gene suppression.

## RESULTS

### PDCD4 expression is regulated by miR-21 and miR-499 in HNSCC.

**FIG 1:**
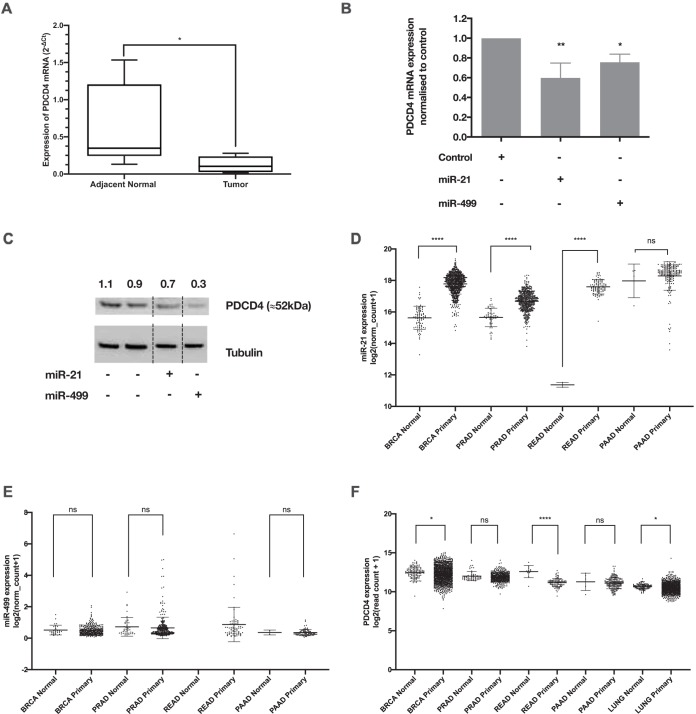
The tumor suppressor gene PDCD4 is downregulated by miR-21 and miR-499. (A) Expression of PDCD4 mRNA in oral cancer tissue versus normal adjacent tissue. The *y* axis represents the fold change of PDCD4 calculated using the 2^−Δ*C_T_*^ method. The box plot represents the interquartile range with the median value. Error bars are standard errors of the means (SEM) (*n* = 6). *, *P* < 0.05 (by Student’s *t* test). (B) Silencing of PDCD4 mRNA in HEK293 cells overexpressing miR-21 or miR-499 mimics. The *y* axis shows PDCD4 mRNA levels normalized to the value for control cells using the 2^−ΔΔ*C_T_*^ method. Error bars are SEM (*n* = 3). *, *P* < 0.05; **, *P* < 0.01; ***, *P* < 0.005 (by one-way ANOVA with a Dunnett test). (C) PDCD4 protein levels in HEK293 cells overexpressing miR-21 and miR-499 mimics. The reductions of PDCD4 protein levels were normalized with the tubulin loading control, and the values for the transfected samples were then normalized to the control. Note that the final image is a composite of various gel lanes. This was required as miR-21 and miR-499 were separated by other samples in the Western blot, as indicated by the dotted lines. (D) Expression of miR-21 within TCGA for a selection of normal and cancer tissues. (E) Expression of miR-499a-5p within TCGA for a selection of normal and cancer tissues. (F) Expression of PDCD4 within TCGA for a selection of normal and cancer tissues. BRCA, breast invasive carcinoma; PRAD, prostate cancer; READ, rectal cancer; PAAD, pancreatic cancer; LUNG, lung cancer; ns, not significant.

In a previous study, we showed that several miRNAs were deregulated in head and neck squamous cell carcinoma (HNSCC) clinical samples and that PDCD4 was regulated in a temporal manner (23). Utilizing a cohort of oropharyngeal SCC clinical samples with adjacent normal tissues (*n* = 6), we confirmed that PDCD4 mRNA is downregulated in cancer tissue (Fig. 1A) and that this regulation is orchestrated by miR-21-5p and miR-499a-5p (Fig. 1B and C). Using The Cancer Genome Atlas (TCGA) database, we also observed an increase in miR-21-5p levels and a decrease in PDCD4 transcripts across several other cancers (Fig. 1D to F).

### PDCD4 is regulated via one miR-21 and two miR-499 target sites.

**FIG 2:**
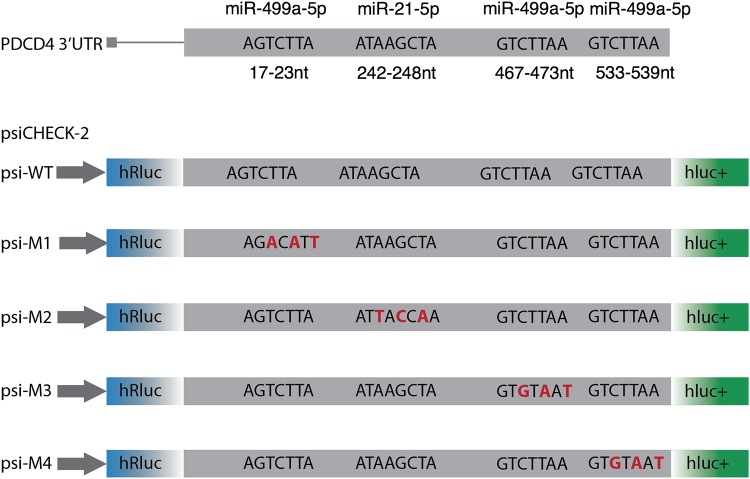
Schematic of the PDCD4 3′ UTR showing the miR-21 and miR-499 sites and their corresponding mutant vectors. Each construct was synthesized using Invitrogen GeneArt and then subcloned into psiCHECK-2. The corresponding mutant vectors were psi-WT for the wild-type 3′ UTR, psi-M1 for mutation of the first miR-499 site, psi-M2 for mutation of the miR-21 site, psi-M3 for mutation of the second miR-499 site, and psi-M4 for mutation of the last miR-499 site. The red nucleotides represent the mutations introduced at the miRNA binding site.

Although both miRNAs regulate PDCD4, the individual contribution of their binding sites to the repression of PDCD4 is unknown. The 3′ UTR of PDCD4 harbors three miRNA sites for miR-499a-5p and one predicted site for miR-21-5p, as predicted by TargetScan (25) (Fig. 2). To assess the individual contribution of these sites to PDCD4 silencing, we synthetized part of the PDCD4 3′ UTR that contains all the studied miRNA target sites. Mutant PDCD4 3′-UTR sequences were developed by modifying the third, fifth, and seventh nucleotides within the miRNA seed region. This was repeated for each studied miRNA site to produce the following four mutant sequences: M2 for the miR-21-5p binding site and M1, M3, and M4 for the three miR-499a-5p binding sites, respectively. These 3′-UTR mutants were then cloned into the dual-luciferase reporter system psiCHECK-2 and transfected into UMSCC22B hypopharyngeal squamous cell carcinoma cells to ascertain the contribution of each individual site to PDCD4 regulation.

**FIG 3:**
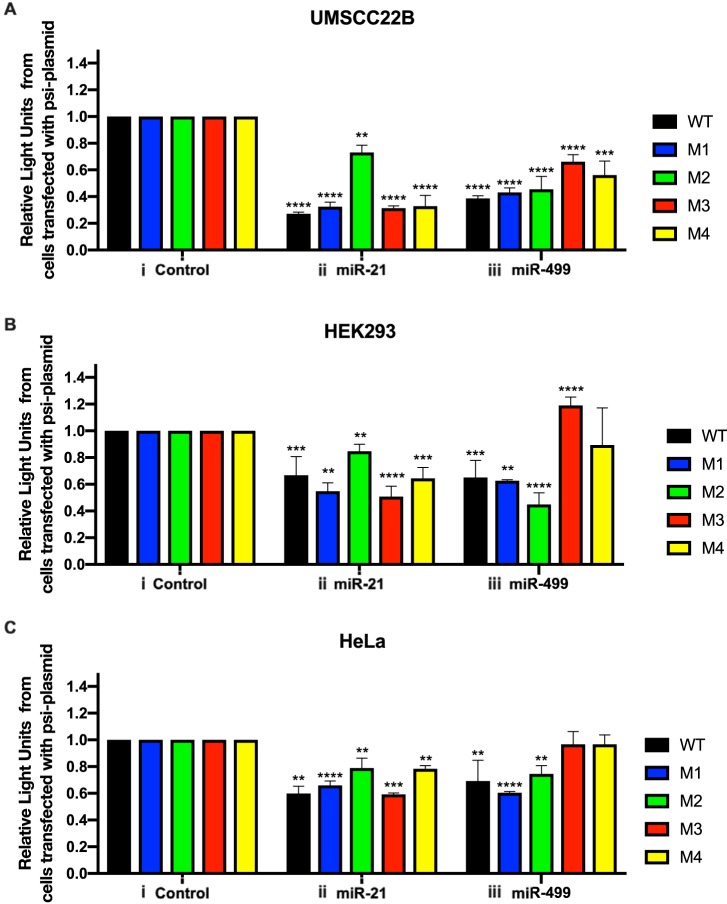
PDCD4 silencing by miR-21 and miR-499a-5p in HEK293 (A), UMSCC22B (B), and HeLa (C) cell lines. Cells were cotransfected with 30 pmol of control (i), miR-21 (ii), and miR-499 (iii) mimics in combination with the psi-WT or mutant vectors. Each color indicates a different psi-CHECK vector. The silencing contribution for each miRNA was assessed using relative light units (luciferase activity) and normalized to the value for control cells. Error bars represent SEM (*n* = 3). *, *P* < 0.05; **, *P* < 0.01; ***, *P* < 0.005 (by one-way ANOVA with a Dunnett test).

To test if the cloned 3′-UTR segment could be targeted by miR-21 and miR-499, we cotransfected the psi-WT reporter, containing the wild-type (WT) PDCD4 3′ UTR, in combination with miR-21 or miR-499 mimics. In UMSCC22B cells, we observed a significant reduction in luciferase activity for cells containing these miRNAs and psi-WT (Fig. 3A). The combination of the psi-M2 mutant and miR-21 resulted in a higher luciferase activity than that of the WT control, indicating the abrogation of miR-21-mediated PDCD4 suppression. However, gene regulation by miR-499 was still observed (Fig. 3Aii).

To determine the contribution of the miR-499 sites, each PDCD4 3′ UTR containing a mutation for miR-499 was individually tested in combination with miR-21 and miR-499 mimics. The cotransfection of miR-499 and psi-M1, containing a mutation in the first miR-499 binding site, resulted in a luciferase activity similar to that of the wild-type 3′ UTR (Fig. 3Aiii). The cotransfection of miR-499 with either the psi-M3 or psi-M4 plasmid resulted in an increase in luciferase activity compared to that of psi-WT (Fig. 3Aiii). Given that one miR-499 site in each of these plasmids is not mutated, it was expected that we would observe a decrease in luciferase activity similar to that of the WT. However, this was not the case, which indicates that both binding sites of miR-499a-5p are necessary for target repression. Therefore, given our results, we suggest that both miR-499a-5p binding sites are required for PDCD4 suppression by miR-499.

This series of transfections was repeated in two independent cell lines, HEK293 and HeLa (Fig. 3B and C). The results from these cell lines demonstrate the same trend as that for UMSCC22B cells, indicating that the underlying mechanism for PDCD4 suppression by miR-21 and miR-499 is present in both cancerous and noncancerous cells. However, the extent of the reduction in luciferase activity of the PDCD4 psiCHECK-2 plasmids is different across the tested cell lines, indicating that the extent of PDCD4 suppression may be cell line specific. From these observations, we concluded that both miR-21 and miR-499 can independently recognize the 3′ UTR of PDCD4 and that the action of miR-499 requires both the second and third miR-499 sites to be intact.

### Effect of miR-21 on efficiency of miR-499 binding of PDCD4.

**FIG 4:**
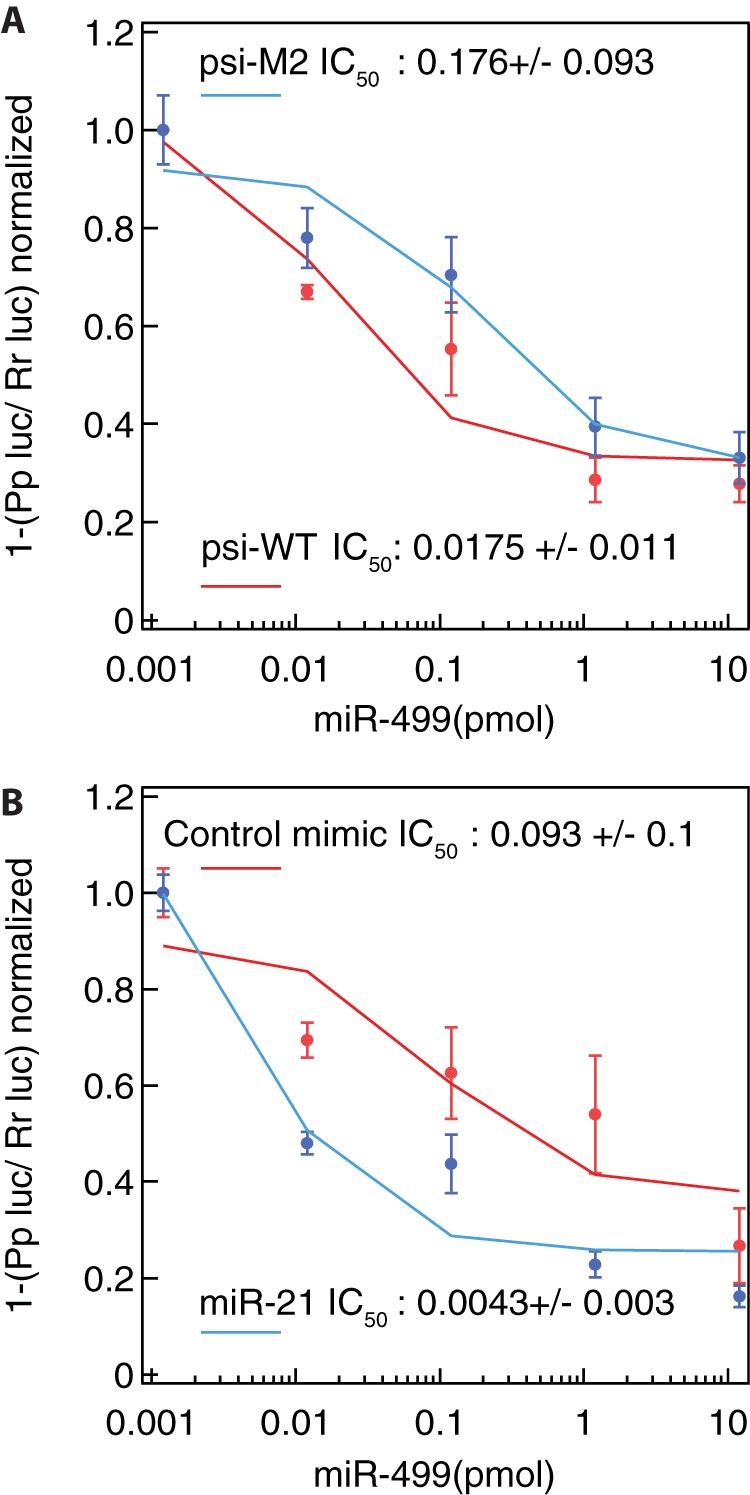
miR-21 increases the silencing potency of miR-499. HEK293 cells were transfected with the miR-499 mimic (concentration range of between 0.0012 and 12 pmol) in combination with either psi-WT or psi-M2. The cells were then harvested at 24 h, and luciferase activity was measured. (A) The silencing potency of miR-499 in cells expressing the miR-21 mutant (psi-M2) and the wild-type (psi-WT) vectors. The calculated IC_50_ for cells harboring the mutant miR-21 vector is 10-fold higher than that for control cells. (B) miR-21 levels can affect miR-499 potency. Cells were transfected with either 30 pmol of the miR-21 or control mimic in combination with the wild-type vector. The addition of miR-21 reduces the IC_50_ required for miR-499 silencing compared to the control mimic. Pp, *Photinus pyralis*; Rr, *Renilla reniformis*.

Although the series of luciferase assays demonstrated that both miR-21 and miR-499 regulate PDCD4, the question remains as to whether its silencing efficiency is altered depending on miRNA abundance and binding site availability. To answer this, we measured the 50% inhibitory concentration (IC_50_) (concentration of miRNA needed to achieve half-maximal silencing of the tumor suppressor PDCD4) of miR-499 by cotransfecting it at increasing concentrations, together with psi-WT or the miR-21 mutant plasmid (psi-M2). The absence of an intact miR-21 binding site increased the IC_50_ by 10-fold compared to that of the wild type. This indicates that more miR-499 is required to suppress the PDCD4 3′ UTR in the absence of the miR-21 site (Fig. 4A).

Next, we asked if miR-21 levels directly influence the efficiency of miR-499 in targeting PDCD4. This is particularly relevant in HNSCC cells, as miR-21 levels are higher than those of miR-499 (23). To test this, cells were cotransfected with psi-WT in combination with either 30 pmol of miR-21 or *let-7* (nontargeting control miRNA mimic). To these same cells, miR-499 was delivered at increasing concentrations. Our results indicated a 21-fold drop in the IC_50_ for miR-499 when miR-21 is expressed at high levels (Fig. 4B). This means that less miR-499 is required to achieve PDCD4 suppression when miR-21 is present at high levels. This therefore could result in a greater suppression of PDCD4 through the action of these two miRNAs.

### miR-21 increases miR-499 levels.

**FIG 5:**
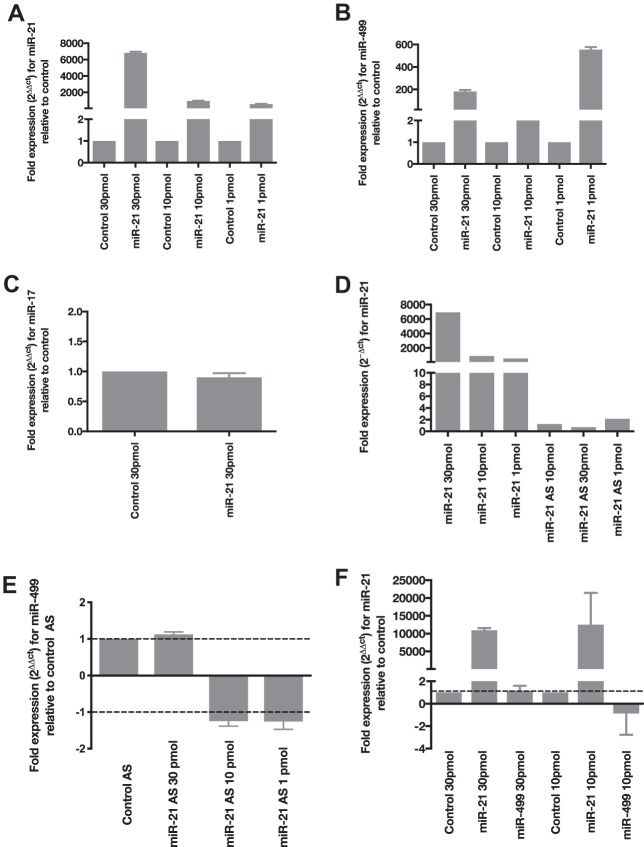
miR-21 increases the expression of miR-499. (A) Expression of miR-21 in cells overexpressing the miRNA mimic. HEK293 cells were transfected with 1, 10, and 30 pmol control or miR-21 mimics. The expression of miR-21 was then normalized to the value for RNU6B, and the resulting fold change is expressed relative to the value for control cells at their specific concentrations. (B) Elevated expression of miR-499 in HEK293 cells transfected with 1, 10, and 30 pmol of the miR-21 mimic. The fold change is expressed relative to the value for control cells at each concentration. (C) Expression of miR-17 is not increased in HEK293 cells overexpressing 30 pmol of the miR-21 mimic. (D) Expression of miR-21 in cells overexpressing the miR-21 mimic or miR-21 antisense (AS). (E) Expression of miR-499 in HEK293 cells transfected with miR-21 AS, delivered at three different concentrations. The fold change in the miR-499 level was normalized to the value for control AS cells at each concentration. (F) Cells overexpressing miR-499 at either 30 or 10 pmol do not show an increase in miR-21 levels. The fold changes of both miRNAs are presented relative to the control (all error bars are SEM [*n* = 3]).

While performing optimizations for the delivery of these miRNAs, we observed that transfection of the miR-21 mimic resulted in an increase in not only the level of miR-21 expression but also the level of miR-499 (Fig. 5A and B). To determine whether miR-21 specifically affected miR-499 levels, we measured the expression level of the control miRNA, miR-17, in the same transfection and observed no change (Fig. 5C). Interestingly, when we transfected miR-21 antisense (AS) to deplete miR-21 levels, there was no change in miR-499 expression (Fig. 5D and E). Although miR-21 increases miR-499 levels, this relationship was not reciprocated, as cells overexpressing miR-499 did not affect the mature levels of miR-21 (Fig. 5F). This suggests that the level of miR-499 is increased only under high-miR-21 conditions and that this relationship is unidirectional.

### miR-21 stabilizes mature miR-499 levels.

**FIG 6:**
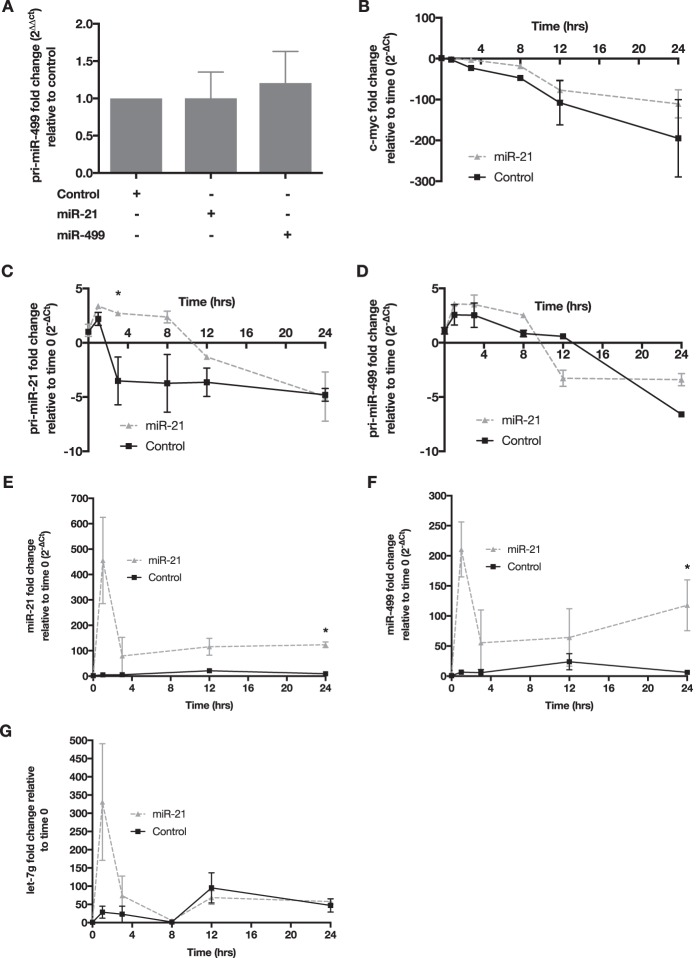
miR-21 stabilizes mature miR-499 levels. (A) pri-miR-499 levels in cells overexpressing either miR-499 or miR-21. The *y* axis represents the fold change of the primary miRNA level, normalized to the value for B2M and then to the value for control cells using the 2^−ΔΔ*C_T_*^ method. (B) Fold changes in c-*myc* mRNA levels in HEK293 cells over 24 h in cells overexpressing miR-21 or control mimics in combination with actD treatment. The expression of c-*myc* was normalized to the value for B2M, and the resulting fold change on the *y* axis was normalized to the value for the control or miR-21 at 0 h. (C) Reduction of the miR-21 primary transcript measured over 24 h in cells overexpressing the miR-21 mimic with actD treatment. (D) Primary miR-499 transcript levels were stabilized in cells overexpressing the mature miR-21 mimic. Fold changes for the primary miRNA transcripts were calculated as described above for c-*myc*. (E) Change in miR-21 levels in actD-treated HEK293 cells overexpressing the miR-21 mimic. (F) Increased expression levels of mature miR-499 over 24 h in actD-treated cells overexpressing miR-21. (G) Expression of Let-7g in actD-treated cells overexpressing miR-21. The mature levels of Let-7g were degraded over the 24-h period. Error bars are SEM (*n* = 3). *, *P* < 0.05 (by a two-sided unpaired *t* test).

To test if miR-21 increased the transcription of miR-499, we quantified the level of primary miR-499 (pri-miR-499) in HEK293 cells that were transfected with either a control, miR-21, or miR-499 mimic. Our data showed no significant changes in the expression levels of pri-miR-499 with miR-21 overexpression (Fig. 6A). Therefore, it was postulated that miR-21 overexpression may elevate the mature levels of miR-499 by affecting its turnover rate. To test this, HEK293 cells were transfected with a control or miR-21 mimic in combination with either actinomycin D (actD), to inhibit *de novo* transcription, or the vehicle control dimethyl sulfoxide (DMSO). To confirm that actD was active in inhibiting transcription, we measured c-*myc*, which has an RNA half-life of approximately 30 min (26). There is a clear reduction of c-*myc* RNA 3 h after actD treatment, indicating that transcriptional activity was effectively inhibited (Fig. 6B). The primary levels of both miR-21 and miR-499 were measured to test our hypothesis that miR-21 affects miR-499 turnover. We observed a rapid decrease in both primary transcripts with miR-21 transfection, indicating that miR-21 and miR-499 transcription was hindered (Fig. 6C and D). As expected, the mature level of miR-21 was elevated due to the transfection of the miR-21 mimic (Fig. 6E). Interestingly, mature miR-499 levels were markedly increased at 24 h, despite transcriptional inhibition by actD (Fig. 6F). To determine whether miR-21 explicitly influences miR-499 processing and stability, we measured the levels of *let-7g* in these samples. We did not observe any increase in *let-7g* levels in miR-21-transfected cells, supporting the specific nature of the relationship between miR-21 and miR-499 (Fig. 6G).

### The expanding regulatory network of miR-21 and miR-499.

Given that miR-21 and miR-499 participate in the coregulation of PDCD4, we postulated that this may be representative of a wider phenomenon in miRNA-mediated silencing. To this end, we analyzed the target prediction results from TargetScan (25) and miRanda (27) for miR-21 and miR-499 and limited the set of predicted genes to those targeted by both miRNAs (Table 1). These targets were then assessed for Ago2 footprints on the 3′ UTR using photoactivatable ribonucleoside-enhanced cross-linking and immunoprecipitation (PAR-CLIP) data (28). Interestingly, the presence of a CLIP-supported miR-21 site on these targets increased the probability of the presence of a downstream predicted CLIP miR-499 binding site by 20%, compared to no CLIP support for the miR-21 binding site. This analysis was expanded by focusing on the common predicted targets of the two miRNAs that contain only one predicted binding site for miR-21 and more than one predicted binding site for miR-499. The probability of having at least one predicted miR-499 site supported by CLIP data increased to 38%. Therefore, a single miR-21 binding site increases the probability of downstream miR-499 binding sites within the same target gene.

**TABLE 1:** Common high-score predictions for miR-21 and miR-499 using TargetScan^*a*^

Gene transcript (GenBank accession no.)	No. of miR-499-5p conserved predicted sites	No. of miR-499-5p poorly conserved predicted sites	Distance(s) between predicted binding sites for miR-499-5p (nt)	Experimental evidence of interaction	
				miR-21	miR-499
SOX6 (NM_001145811)	5	1	170, 305, 15, 287, 290	NA	Luciferase
PURB (NM_033224)	1	3	1,726, 3,430, 750	Microarray	NA
PDCD4 (NM_001199492)	3	0	450, 66	Luciferase, Western blotting	Luciferase, qRT-PCR, Western blotting
JHDM1D (NM_030647)	1	2	4,229, 7	NA	NA
SECISBP2L (NM_001193489)	1	2	1,080, 1,428	NA	NA
PAIP2B (NM_020459)	1	2	729, 52	NA	NA
SOX5 (NM_006940)	2	0	158	Western blotting, Northern blotting, qRT-PCR, luciferase	NA
SCRN1 (NM_001145513)	1	1	3,334	Microarray	NA
ARHGAP32 (NM_001142685)	1	1	49	NA	NA
KLHDC5 (NM_020782)	1	1	2,175	NA	NA
RAB22A (NM_020673)	1	1	1,650	NA	NA
MEIS1 (NM_002398)	2	0	461	Microarray	NA
LRP6 (NM_002336)	1	1	576	NA	NA
FOXP2 (NM_001172766)	1	1	26	NA	NA
STRN (NM_003162)	1	1	1,853	NA	NA

a Each transcript in the table represents at least one conserved binding site predicted for miR-21-5p and at least two binding sites predicted for miR-499a-5p. SOX6, SRY box 6; NA, not applicable.

Next, we put forward the notion that miR-21 may promote cotargeting with other downstream miRNA sites besides miR-499. To test this, we randomly selected 10 other miRNAs from a shuffled pool of the miRNAs present in miRBase version 20 (29). The above-described analysis was applied to these randomly selected miRNAs, using the prerequisite of containing a single miR-21 upstream site. A list of overlapping genes was generated for miR-21 and each of the randomly selected miRNAs. We then inspected if the presence of a single CLIP-supported predicted binding site for miR-21 increased the probability of having more than one CLIP-supported predicted binding site for the randomly chosen miRNAs. For all 10 selected miRNAs, we observed a significant increase (*P* < 0.0001 by Bernard’s exact test) in the probability of having at least one CLIP-supported predicted binding site (Table 2). In other words, there is a strong statistical likelihood that the presence of a single upstream miR-21 binding site will result in an adjoining downstream miRNA binding site.

**TABLE 2:** CLIP-Seq data showing miR-499 and miR-21 interaction with mRNA targets^*a*^

Gene transcript (GenBank accession no.)	No. of CLIP-Seq experiments supporting miR-21 interaction with the predicted target	No. of CLIP-Seq experiments supporting miR-499 interaction with the predicted target
SOX6 (NM_001145811)	*	*
PURB (NM_033224)	3	*
PDCD4 (NM_001199492)	12	1
JHDM1D (NM_030647)	3	3
SECISBP2L (NM_001193489)	*	1
PAIP2B (NM_020459)	*	*
SOX5 (NM_006940)	9	2
SCRN1 (NM_001145513)	1	6
ARHGAP32 (NM_001142685)	4	*
KLHDC5 (NM_020782)	8	2
RAB22A (NM_020673)	1	3
MEIS1 (NM_002398)	9	1
LRP6 (NM_002336)	10	0
FOXP2 (NM_001172766)	9	*
STRN (NM_003162)	*	*

a * indicates the absence of cross-linking immunoprecipitation sequencing (CLIP-Seq) evidence of the interaction of the corresponding miRNA and its predicted target.

**FIG 7:**
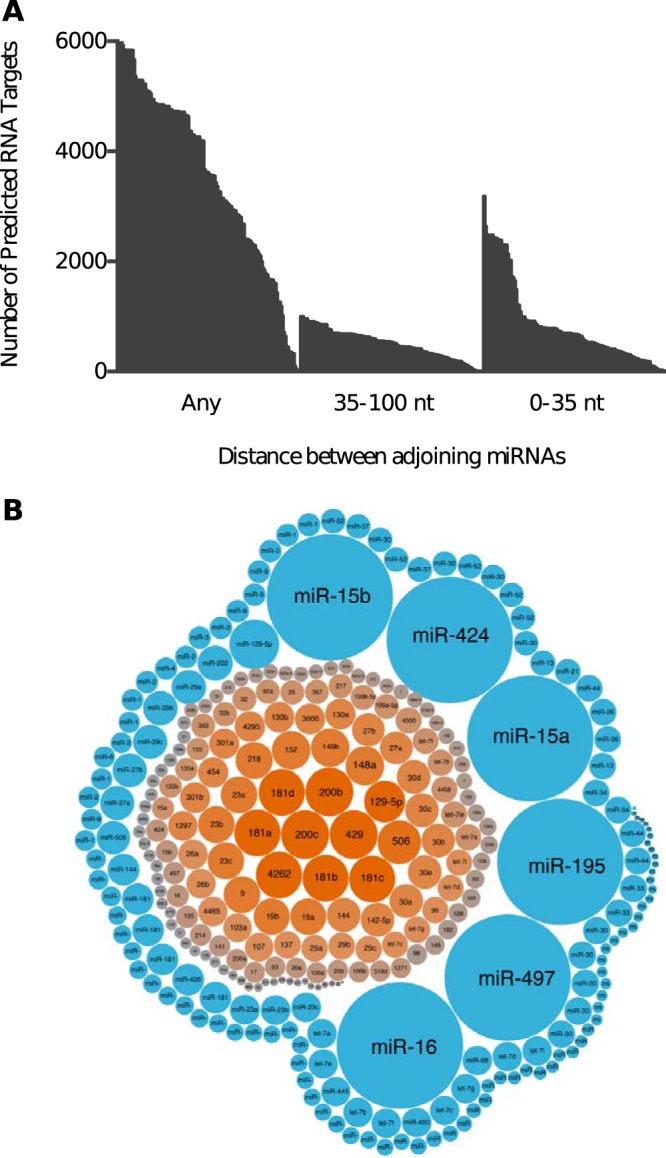
Adjoining miRNA sites separated by 35 to 100 nt are overrepresented on target 3′ UTRs. (A) Distribution plot showing the number of potential targets for 167 adjoining miRNA sites separated by 35 to 100 nt or 0 to 35 nt or with no distance constraints (Any). (B) Bubble chart showing the specific miRNAs that were overrepresented as adjoining pairs separated by either 35 to 100 nt (orange) or 0 to 35 nt (blue). The larger bubble size indicates a greater number of targets with adjoining sites for the specific miRNA.

Finally, as the two miR-499 sites were separated by 66 nt and were both required for PDCD4 suppression, we investigated if there was an overrepresentation of other adjoining miRNA sites separated by similar distances. The subsequent analysis was restricted to 163 conserved miRNAs with the distance between adjoining pairs set between 35 and 100 nt. This threshold was set as previous reports have stated that distances of less than 35 nt have a cooperative effect (11–14). In total, there were 96,000 3′ UTRs with adjoining miRNA binding sites separated by 35 to 100 nt. In comparison, over 165,000 3′ UTRs were targeted by adjoining miRNAs less than 35 nt apart (Fig. 7A). This suggests that adjoining miRNA pairs separated by 35 to 100 nt may have a wider role in gene regulation. We then sought to identify which adjoining miRNA pairs were the most prominent in the 35- to 100-nt and <35-nt data sets. The analysis of the 35- to 100-nt data set indicated that miR-200c, miR-200b, and miR-429 adjoining pairs were overrepresented on more than 2,300 3′ UTRs. In contrast, miR-16, miR-497, miR-195, miR-15a, miR-15b, and miR-424 pairs were found on 16,000 3′ UTRs in the <35-nt data set (Fig. 7B). This suggests that the miRNAs in adjoining pairs separated by larger distances are distinct from those in adjoining pairs separated by 35 nt or less.

## DISCUSSION

PDCD4 is a translational inhibitor which is often upregulated during apoptosis (30). However, in a disease setting such as cancer, PDCD4 is frequently downregulated, and its expression has been correlated with disease progression in various malignancies (2, 31, 32). We and others have further shown that the PDCD4 protein is deregulated in oral tumors and is posttranscriptionally regulated by miR-21 and miR-499 (18, 23). On this basis, we investigated whether there was a relationship between these two miRNAs in their control of PDCD4.

To understand the individual involvement of miR-21 and miR-499 in the silencing of PDCD4, we mutated the miR-21 and three miR-499 binding sites within its 3′ UTR and tested their contribution using a series of luciferase assays. As expected, mutations at the miR-21 seed region abolished miR-21-mediated silencing, which is congruent with other studies (33). Our results indicate that the first miR-499-5p binding site does not contribute to PDCD4 suppression, likely due to the impedance of ribosomes within the first 15 nt of the 3′ UTR (34).

To our surprise, the second and third miR-499 binding sites appear to be dependent. In the series of luciferase assays across several cell lines, the mutation of one of these sites abolished PDCD4 silencing, despite the presence of a nonmutated site. This finding is novel and is distinct from the cooperative action observed for adjoining miRNA sites. These two miR-499 sites are 66 nt apart and are therefore beyond the 13- to 35-nt distance that defines miRNA site cooperativity (11–13). It has been reported that miRNA-loaded Ago2 scans the target RNA by lateral diffusion and binds to complementary regions matching the seed (35). It has also been demonstrated that neighboring miRNA binding sites synergistically increase the dwell time of Ago2 on target RNAs. Perhaps both miR-499 sites are needed to establish the required dwell time on PDCD4 for Ago2-bound miR-499-mediated silencing.

Our results also indicate that the efficiency of miR-499 targeting is increased by the presence of both an intact miR-21 binding site and high levels of miR-21. This may be explained by the thermodynamic properties of their respective binding sites. Within the RNA secondary structure, the miR-21 binding site has a lower free energy, Δ*G*, of −2.69 and thus is the most energy-efficient site for the access of the RNA-induced silencing complex (RISC). The first miR-499 binding site has the highest free energy (Δ*G* of 6.04) and is the least accessible to the RISC, as discussed above. The Δ*G* values of the last two binding miR-499 sites are −1.57 and −1.58, respectively (23). Based on the thermodynamic properties of these binding sites, we reasoned that in a cellular environment with high miR-21 levels, the loaded miR-21–Ago2 complex first binds to the more accessible miR-21 site within PDCD4. This initial event may induce conformational changes in the tertiary structure of the PDCD4 3′ UTR to increase the accessibility of the distal sites, allowing for the efficient binding of Ago2-bound miR-499. This may provide an explanation as to how miR-21 increases miR-499 binding efficiency, as observed in our results.

The steady-state levels for most miRNAs require a balance between transcription and degradation (36); our results indicate that miR-21 stabilizes mature miR-499 but does not promote the synthesis of new primary miR-499. This example of miRNA-mediated miRNA regulation is in contrast with those studies showing a direct binding between two miRNAs (37) or an influence on miRNA abundance through the biogenesis pathway (38). In melanoma, lung, and breast cancer cell lines, it was demonstrated that miR-107 could directly interact with Let-7 to promote its degradation (37). Additionally, miR-709 has been shown to bind to a 19-nt region within the primary transcript of miR-15a/16 to abrogate downstream maturation (39). More recently, miR-21 was shown in colon cancer stem cells to reduce the expression of miR-145 (40). These examples all highlight a negative regulatory role for miRNAs in the control of other miRNAs. However, our study demonstrated a positive regulatory role for miR-21 in the modulation of miR-499.

The key question that arose during our investigations was in regard to the stabilization of mature miR-499, for which we suggest two possible explanations. The first is that these two miRNAs can bind to each other to form hybrid duplexes (41). Using miRWalk 2.0 to examine possible hybridization patterns between different miRNAs, we observed that the only miR-21-5p binding partner, from a pool of 1,880 miRNAs, was miR-499-3p. Thus, it may be that the 5p arm of miR-21 interacts with the 3p arm of miR-499 to increase the levels of miR-499-5p. Another scenario is that the overexpression of miR-21 affects the target pool of miR-499. This was first reported in Caenorhabditis elegans (42), whereby the overexpression of an artificial target bearing three *let-7* binding sites increased *let-7* levels. Conversely, a reduction in target availability reduces mature miRNA levels (42). Another recent study using mammalian cells found that decay rates for miRNAs can be influenced by target abundance (43). This is speculation, but perhaps the overexpression of miR-21 creates a larger pool of miR-499 targets, allowing for the stabilization of miR-499 levels (44).

Given that miR-21 is one the highest-expressing miRNAs in most cancers and diseased tissue, we suggest that miR-499 is not the only recipient of this positive regulation. Our computational results indicate that the presence of a miR-21 binding site increased the likelihood of adjoining downstream miRNA binding sites. Therefore, this mode of regulation is not restricted to miR-499 but may represent an additional layer of miRNA control. This bioinformatic evidence suggests a far greater regulatory potential for miR-21 in conjunction with adjoining miRNA pairs and the wider influence of these interactions on the microRNAome.

To conclude, we show that PDCD4, a tumor suppressor gene suppressed in many cancers, is regulated through a relationship between miR-21 and miR-499. We demonstrate that the first miR-499 binding site on PDCD4 is not active, but the remaining two miR-499 sites are both needed to elicit miR-499-mediated silencing. Additionally, the binding of miR-21 amplified the silencing potency of miR-499 by 10-fold, and miR-21 appears to stabilize the levels of mature miR-499. As miR-21 is a prominent oncomiR (45, 46), we show that it may have an additional role in increasing the binding efficiency of lowly expressed miRNAs. Also, the presence of a miR-21 site increases the probability of downstream adjoining miRNA sites, indicating a suggested role for miR-21 cooperation with other miRNAs. Our results highlight the complexity of miRNA regulation far beyond the simplistic model of one miRNA to one target gene. The regulation of PDCD4 by miR-21 and miR-499 typifies the complex partnerships that can exist between regulatory miRNAs when targeting mammalian tumor suppressor genes.

## MATERIALS AND METHODS

### Patient samples and cell lines.

Head and neck tumor tissue and microscopically matched normal adjacent tissue (2 cm outside the surgical margin) (*n* = 6) were used for quantitative real-time PCR (qRT-PCR) analysis. Usage of these patient samples was approved by the Research Ethics Committee at Royal Prince Alfred Hospital, Sydney, Australia (protocols X05-269 and X05-270). This protocol covered consent and collection of material excess to diagnostic requirements for research purposes only.

### TCGA analysis.

Gene and miRNA expression data, and associated clinical data for various cancers from The Cancer Genome Atlas (TCGA), were downloaded from the UCSC Xena Functional Genomics Explorer (https://xenabrowser.net). The data were filtered to separate the expression levels for primary tumor and matched normal samples. miR-21, miR-499, and PDCD4 expression was plotted and analyzed using GraphPad Prism 8.0.1.

### Cell culture.

HEK293, HeLa, and UMSCC22B cell lines (Invitrogen) were grown in Dulbecco’s modified Eagle’s medium (DMEM) (4.5 g/liter glucose, 4 mM l-glutamine, and 110 mg/liter sodium pyruvate) (Life Technologies), supplemented with 10% fetal bovine serum (Gibco) at 37°C with 5% CO_2_.

### Isolation of RNA from fresh tissue or cultured cells.

Approximately 100 mg of fresh frozen tissue was diced, homogenized, and then rinsed with 1 ml of TRIzol reagent (Invitrogen). For cell lines, 1 ml of TRIzol was added to the cell pellet, with further disruption through a 21-gauge needle. Total RNA was then extracted using isopropanol precipitation and quantified using a NanoDrop ND1000 instrument (Thermo Fisher Scientific). Samples with a 260/280-nm absorbance ratio in the range of 1.71 to 2.1 were used for downstream studies.

### DNA and RNA transfection.

Lipofectamine 2000 was used to transfect plasmid DNA (50 ng) and miRNA mimics (Ambion) into HEK293, UMSCC22B, and HeLa cells. According to the manufacturer’s instructions, 4 × 10^4^ cells were seeded into each well of a 24-well plate prior to transfection. DNA was diluted with Opti-MEM (Life Technologies), while 1.5 μl Lipofectamine 2000 was diluted with Opti-MEM and incubated for 5 min before both diluents were combined. The mixture was incubated for 20 min at room temperature and then added to each well of a 24-well plate. After transfection, cells were harvested, and luciferase activity was measured using the dual-luciferase reporter assay system (Promega).

Alternatively, we also utilized Lipofectamine RNAiMAX (Invitrogen) to deliver miR-21, miR-499, a scrambled precursor, and *let-7a* (negative control) into HEK293 cells. Briefly, 1 × 10^5^ cells were seeded into wells of a 6-well plate. miRNA mimics at various concentrations (1 pmol, 10 pmol, and 30 pmol) and Lipofectamine RNAiMAX were diluted with Opti-MEM separately for 5 min and then incubated together for 20 min before addition to cells. Total RNA was collected 24 h after transfection and used for downstream molecular analysis.

### Quantitative real-time PCR.

miRNA and mRNA expression levels were measured using the Applied Biosystems StepOne real-time PCR systems machine and TaqMan assays (Life Technologies). RNU6B or U6 small RNA was used for the normalization of miRNA expression, while glyceraldehyde-3-phosphate dehydrogenase (GAPDH) or actin acted as the reference/calibrator control. The fold expression of these RNAs was calculated using the 2^−ΔΔ*C_T_*^ method (47). For fold change values of between 0 and 1, the negative inverse (−1 divided by the calculated fold change) was calculated to better visualize and interpret the results.

### Cloning of wild-type PDCD4 and mutant 3′ UTRs into luciferase reporter vectors.

The wild-type and mutant 3′ UTRs were synthesized by GeneArt (Invitrogen) and cloned into the multiple-cloning site (MCS) of the psiCHECK-2 vector (Promega) using the restriction enzymes EagI and NotI. The expression of the *Renilla* signal was used to determine the regulation of the PDCD4 3′ UTR by miRNA mimics. The firefly luciferase gene in psiCHECK-2 was used for normalization. The psi-M2 construct contained the 3′-UTR PDCD4 sequence with single nucleotide mutations at the 3rd, 5th, and 7th nucleotides at the miR-21 seed sequence (nt 242 to 249). This same principle was applied to the psi-M1 construct with mutations at the first miR-499 seed sequence (nt 17 to 23), psi-M3 with mutations at the second miR-499 seed sequence (nt 467 to 473), and psi-M4 with mutations at the third miR-499 seed sequence (nt 533 to 539).

### Measurement of protein expression by Western blotting.

Transfected cells were harvested and lysed with CellLytic M cell lysis reagent (Sigma-Aldrich) supplemented with 0.1% serine protease inhibitor (Sigma-Aldrich). The protein concentration was determined using the Bradford assay (Bio-Rad) and the Qubit 2.0 fluorometer (Life Technologies), with bovine serum albumin (BSA) acting as a standard. Twenty micrograms of protein was loaded onto a precast NuPAGE (4 to 12%) Bis-Tris gel (Invitrogen) in NuPAGE morpholineethanesulfonic acid (MES)-SDS running buffer (50 mM MES, 50 mM Tris base, 0.1% SDS, 1 mM EDTA). After electrophoresis, the proteins were transferred onto a polyvinylidene difluoride (PVDF) membrane (Pierce) using NuPAGE transfer buffer (25 mM Bicine, 25 mM Bis-Tris, 1 mM EDTA). The membrane was incubated with PDCD4 (1:5,000) or tubulin (1:1,000), followed by conjugation with a secondary antibody containing horseradish peroxidase (HRP). Chemiluminescence with the ECL plus reagent (GE Healthcare Life Sciences) was used to visualize the specific protein bands.

### Measurement of miRNA inhibition by luciferase assays.

The Dual-Luciferase reporter assay system (Promega) was used to measure the luciferase activity of cells cotransfected with wild-type and mutant PDCD4 3′-UTR vectors in combination with miRNA mimics. Firefly and luciferase activities were measured 24 h after transfection. The cells were washed with a phosphate-buffered saline (PBS) solution and lysed with 100 μl of passive lysis buffer (Promega) for 15 min at room temperature. The contents of each well were then freeze-thawed to complete lysis, and 20 μl of the lysate was added to individual wells of an opaque 96-well plate (PerkinElmer). This was followed by the addition of 100 μl of luciferase assay reagent II (LARII) (Promega). The Tecan Infinite 200 Pro spectrophotometer was then used to measure firefly activity. After this measurement, 100 μl of Stop & Glo reagent (Promega) was added to each well, and *Renilla* activity was measured. For each luminescence reading, there was a 2-s premeasurement delay, followed by a 10-s measurement period. Analyses of all samples were performed in triplicates, and luciferase activity was expressed as a *Renilla*/firefly ratio to normalize for cell number and transfection efficiency. Each transfection condition was calculated as a ratio of the control *let-7a* luciferase activity. This calculation was separately applied to each of the different mutant transfection conditions.

### Cotransfection of reporter mutant vectors at various miRNA mimic concentrations.

To evaluate the contribution of these miRNA sites to silencing efficacy, we cotransfected miRNA mimics at various concentrations (0.0012, 0.012, 0.06, 0.12, 1.2, and 12 pmol) in combination with the WT or mutant vectors. These miR-21 or miR-499 mimics were mixed with 50 ng the 3′-UTR PDCD4 vector and transfected into 4 × 10^4^ HEK293 cells in a 12-well plate. The cells were then harvested at 24 h, and luciferase activity was measured. The IC_50_ was calculated using the Hill equation (48), and propagated error and titration graphs were created with the Igor Pro Carbon program.

### Quantification of miRNA stability using actinomycin D.

A total of 4 × 10^5^ cells were seeded into a 6-well plate. After 24 h, cells were transfected with control miRNA and miR-21 mimics at 30 pmol and incubated for 3 h before the addition of actinomycin D (actD) (5 μg/ml). After the addition of actD, cells were lysed with RNAzol at the 0-, 1-, 3-, 12-, and 24-h time points, and RNA was isolated for subsequent quantitative PCRs (qPCRs) measuring miRNA stability. The turnover rate for each miRNA was calculated using the 2^−ΔΔ*C_T_*^ method to determine the fold change of the miRNA with overexpression of the control mimic and miR-21 in cells. The fold change of the miRNA at each time point was then normalized to the value at time zero.

### Bioinformatics analysis.

Predicted targets of miR-21, miR-499, and randomly selected miRNAs used in this study were downloaded from TargetScan version 7.0 and miRanda, August 2010 release. The random selection of miRNAs from the shuffled pool was based on the condition that no selected miRNA would share the same seed region as miR-21. To achieve this, all miRNAs with the same seed region as miR-21 were removed from the miRNA pool. Both miRanda and TargetScan were set at a low prediction cutoff to obtain the maximum number of target predictions for each miRNA. The preprocessed PAR-CLIP data set of genome-wide Ago2 interactions was downloaded from StarBase v2.0. Locus site coordinate mapping of the predicted binding sites of miRNAs and the Ago2 footprints was performed in Python. Predicted binding sites for miRNAs with Ago2 interaction support were regarded as those with an overlapping length of at least 1 nucleotide and a colocalized Ago2 footprint on the same loci. Alternatively, miRNA binding sites without Ago2 interaction support were classified as those devoid of an Ago2 CLIP site on the same loci.

### Statistical analysis.

One-way analysis of variance (ANOVA) with Dunnett’s multiple-comparison test was used to analyze the series of luciferase assays. Fold change values were analyzed using unpaired two-tailed Student’s *t* test. All *P* values of <0.05 were considered statistically significant. All statistical calculations were performed in Prism version 8 (GraphPad).
